# FBStrNet: Automatic Fetal Brain Structure Detection in Early Pregnancy Ultrasound Images

**DOI:** 10.3390/s25165034

**Published:** 2025-08-13

**Authors:** Yirong Lin, Shunlan Liu, Zhonghua Liu, Yuling Fan, Peizhong Liu, Xu Guo

**Affiliations:** 1 School of Medicine, Huaqiao University, Quanzhou 362021, China; linyirong2000@126.com; 2 Department of Ultrasound, The Second Affiliated Hospital of Fujian Medical University, Quanzhou 362000, China; 85443785@fjmu.edu.cn; 3 Department of Ultrasound, Quanzhou First Hospital Affiliated to Fujian Medical University, Quanzhou 362000, China; liuzhonghua2005@126.com; 4 College of Engineering, Huaqiao University, Quanzhou 362021, China; yling_fan@126.com

**Keywords:** deep learning, early pregnancy, fetal brain, ultrasound imaging, anatomical structure detection

## Abstract

Ultrasound imaging is widely used in early pregnancy to screen for fetal brain anomalies. However, the accuracy of diagnosis can be influenced by various factors, including the sonographer’s experience and environmental conditions. To address these limitations, advanced methods are needed to enhance the efficiency and reliability of fetal anomaly screening. In this study, we propose a novel approach based on a Fetal Brain Structures Detection Network (FBStrNet) for identifying key anatomical structures in fetal brain ultrasound images. Specifically, FBStrNet builds on the YOLOv5 baseline model, incorporating a lightweight backbone to reduce model parameters, replacing the loss function, and utilizing a decoupled detection header to improve accuracy. Additionally, our method integrates prior clinical knowledge to minimize false detection rates. Experimental results demonstrate that FBStrNet outperforms state-of-the-art methods, achieving real-time detection of fetal brain anatomical structures with an inference time of just 11.5 ms. This capability enables sonographers to efficiently visualize critical anatomical features, thereby improving diagnostic precision and streamlining clinical workflows.

## 1. Introduction

The first-trimester fetal ultrasound scan is a critical component of obstetric care, aimed at assessing fetal viability and evaluating fetal anatomy for the early detection of structural anomalies [1,2]. Anomalies that should always be detected in a first-trimester fetal ultrasound scan include body stalk anomaly, anencephaly, alobar holoprosencephaly, exomphalos, gastroschisis, and megacystis [3]. The International Society of Ultrasound in Obstetrics and Gynecology (ISUOG) clinical protocol for the first trimester scan defines these key tasks and measurements, carried out between 11^+0^ and 14^+0^ weeks of gestation. According to the ISUOG guidelines, examination of the fetal head and central nervous system is best achieved using a combination of axial and midsagittal planes [2]. According to ISUOG guidelines, we selected four fetal brain ultrasound (FBUS) images to assess fetal brain anatomy, such as the trans-lateral ventricular axial plane (TLVAP), trans-posterior fossa axial plane (TPFAP), trans-thalamic axial plane (TTAP), and mid-sagittal plane (MSP). The four planes make it possible to clearly observe the calcification of the skull. The fetal brain’s various anatomical structures, such as the cranial bone (CB), brain midline (BM), choroid plexus (CP), aqueduct of Sylvius (AS), thalamus and cerebral peduncles (T&P), cisterna magna (CM), brainstem (B), fourth ventricle (FV), nasal bone (NB), mandible (M), third ventricle (TV), hard palate (HP), and other important areas, can be seen. This enables the doctor to conduct a thorough anatomical and physiological evaluation of the fetal brain.

Ultrasound imaging is a widely utilized tool for screening fetal brain anomalies during early pregnancy [4]. However, several unavoidable factors can influence the accuracy of a sonographer’s diagnosis. First, technical limitations such as the inherent inaccuracy of ultrasound imaging, low signal-to-noise ratios, and the presence of speckle noise often compromise the clarity of the images. Second, the diagnostic outcome is heavily dependent on the operator’s skill, including their proficiency in ultrasound operation, adherence to standardized screening protocols, and ability to accurately identify fetal brain anatomical structures [5]. Additionally, external factors such as the equipment model, the small size of fetal organs in early pregnancy, and the fetal position during the examination can further impact the completeness and accuracy of the anatomical visualization [6]. Given these challenges, there is a pressing need to develop more effective methods to enhance the screening and diagnostic efficiency of sonographers, thereby improving the detection of fetal malformations and contributing to the goals of eugenics.

Deep learning developments in recent years have yielded innovative solutions for FBUS analysis. Although convolutional neural networks (CNNs), such as YOLOv3 [7] and YOLOv5 [8], have demonstrated increased accuracy in adult ultrasound tasks, ultrasound-specific noise and the small size of anatomical targets during early gestation continue to restrict their performance in FBUS. Transformer-based architectures employ self-attention mechanisms to introduce global context modeling, but their quadratic computational complexity renders them difficult to apply in real-world scenarios. Lightweight designs of domain-specific adaptations like MobileNetV2 [9] and EfficientNetV2 [10] help to alleviate these problems to some degree, but they still struggle to strike a balance between bounding box precision and classification accuracy when facing FBUS constraints. Our proposal is the Fetal Brain Structures Detection Network (FBStrNet), a specialized framework for accurate, real-time key anatomical structures (KASs) localization in early pregnancy FBUS to overcome this technical bottleneck. In summary, the key contributions of this paper are as follows:

(1) This study addresses the challenge of automatic detection of KASs in FBUS images through FBStrNet, an innovative multi-task deep learning framework.
(2) By integrating YOLOv5 with a feature fusion strategy, FBStrNet achieves unprecedented precision in detecting 12 critical anatomical structures, significantly improving both detection accuracy and computational efficiency.
(3) Experimental results demonstrate the model’s exceptional robustness under low signal-to-noise ratio conditions, making it a valuable assistive tool for sonographers to identify key structures in real-time, thereby enhancing the efficiency and accuracy of fetal brain development assessments.

## 2. Related Work

With the progress of deep learning, the application of these methods for medical image analysis has increased. Several clinical issues have been solved, improving the accuracy of clinical diagnoses and reducing the burden on sonographers. Among the most advanced and wide applications is the recognition of medical images.

Yaqub et al. [5] employed deep learning techniques to achieve precise fetal brain localization, enabling detection of the cavum septi pellucidi (CSP) and accurate identification of the trans-ventricular axial plane (TVAP). Their framework facilitates comprehensive fetal brain symmetry assessment, providing clinically actionable insights for neurodevelopmental evaluation. Lin et al. [11] designed a Mask Focal R-CNN (MF R-CNN) to detect six anatomical structures: lateral sulcus, thalamus, CP, CSP, TV, and BM. By integrating image magnification with clinical protocols, their method enhances TTAP, standardization, and ultrasound image quality assessment. Zhang et al. [12] proposed a multi-task model for automated quality assessment of fetal head, abdomen, and cardiac ultrasound images. By detecting anatomical structures to determine compliance with clinical standards, their approach achieves state-of-the-art performance in both segmentation accuracy and diagnostic reliability. Pu et al. [13] developed a hybrid CNN-RNN architecture for fetal standard plane recognition in industrial IoT environments. The CNN identifies anatomical structures and classifies four planes (TTAP, Trans-cerebellar Axial Plane (TCAP), abdomen, and lumbosacral vertebrae plane), while the RNN captures temporal dependencies for organ tracking across video frames. Coronado-Gutierrez et al. [14] implemented a deep learning system to identify TTAP, TVAP, and TCAP planes. Their framework automatically segments nine anatomical structures, including CSP, anterior horn superior/inferior, thalamus superior/inferior, Sylvian fissure, interhemispheric fissure, and cerebellum, while performing automated biometric measurements. Guo et al. [15] introduced a coarse-to-fine detection framework combined with multi-task learning to optimize ultrasound standard plane acquisition. Their feature fusion strategy significantly improves detection efficiency and accuracy across multi-scale images. Han et al. [16] created CUPID, an automated software for standard plane recognition (TCAP, TTAP, TVAP) and fetal biometric measurement. This tool enhances clinical workflows through efficient detection of anatomical structures and quantitative assessment of fetal brain development.

Automatic recognition and detection of FBUS images have been the focus of extensive research in recent years, with most studies concentrating on fetal ultrasound images from 18 to 40 weeks of gestation. In contrast, first-trimester fetal ultrasound images have received limited attention. Existing research in this area has primarily focused on identifying fetal images with normal nuchal translucency [17], evaluating fetal health [18], and automating the segmentation of video clips containing anatomical structures [1]. However, there is a significant gap in research on the automatic recognition of key early-pregnancy ultrasound planes, including the TLVAP, TPFAP, TTAP, and MSP, as well as the critical anatomical structures visible in these planes. The model developed in this study aims to address this gap by assisting diagnostic sonographers in rapidly acquiring FBUS images for use as a teaching tool and for ultrasound didactic training. Additionally, it will aid in the identification of important anatomical structures within FBUS images.

## 3. Methodology

### 3.1. Model Framework

**Figure 1:**
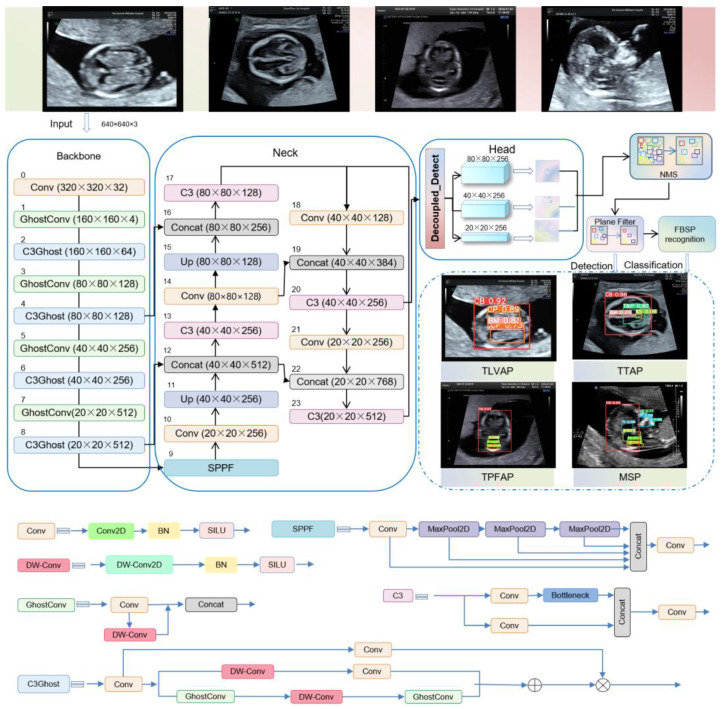
The structure of the FBStrNet model.

In this study, we established YOLOv5 as the baseline architecture and developed FBStrNet, a novel deep learning framework specifically designed for detecting twelve critical anatomical structures in FBUS images. As depicted in Figure 1, FBStrNet is structured around three synergistic components: (1) a lightweight backbone network optimized for efficient feature extraction, (2) a hierarchical interaction module designed to integrate multi-scale contextual information, and (3) a decoupled prediction head tailored for precise localization and classification of anatomical structures. Owing to their critical roles in model efficacy, we now provide an in-depth analysis of the backbone network and the decoupled prediction head, elucidating their architectural innovations and functional contributions.

**Figure 2:**
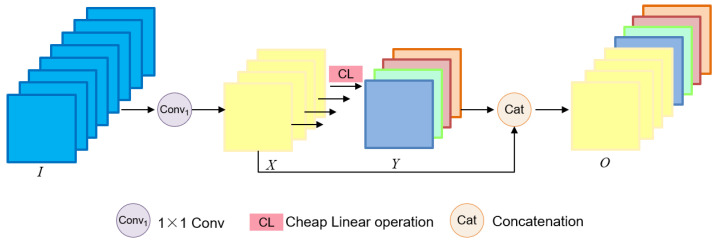
Ghost module.

To balance model efficiency and detection accuracy, we focused on optimizing the backbone architecture. Specifically, we integrated the Ghost module [19] into its backbone architecture. In the Ghost module, the input (I) is processed through a 1 × 1 convolution to produce many intrinsic features (X) and reduce the feature channel to half its original number. Then, each intrinsic feature is convolved by a deep convolution of the Cheap Linear Operations (CL) to produce a ghost feature (Y). The concatenation (Cat) of intrinsic and ghost features yields the final feature map, which is the output (O). Figure 2 demonstrates the Ghost module’s mechanism of function. Through a decrease in convolution kernel count and an increase in linear operations, the Ghost module effectively decreases network complexity. We optimized the Conv and C3 modules as the GhostConv and C3Ghost modules, respectively, and introduced the Ghost module to YOLOv5. The deep convolution is employed in the GhostConv module to create extra features through low-cost linear operations. Figure 1 describes the structure of the GhostConv module and the C3Ghost module in FBStrNet. Replaced the C3 module in Layers 1, 3, 5, and 7 with the C3Ghost module, and the Conv module in Layers 2, 4, 6, and 8 with the GhostConv module; the stride size of Conv2D is 2.

**Figure 3:**
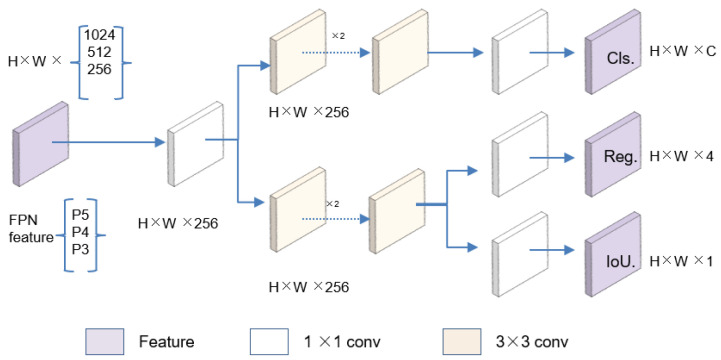
The module of the Decoupled_Detect.

After optimizing the backbone architecture, we turned our attention to the detection head, which plays a vital role in accurately identifying and locating the target anatomical structures. To enhance the detection performance, we introduced Decoupled_Detect. It serves as a decoupling head [20], extracting the target location and category information, learning these aspects separately through distinct network branches, and ultimately fusing them. Specifically, each layer of FPN features initially employs a 1 × 1 convolutional layer to diminish the number of feature channels to 256. Subsequently, it integrates two parallel branches, each of which consists of two stacked 3 × 3 convolutional layers and is dedicated to classification and regression tasks, respectively. An IoU branch is appended to the regression branch. In the regression branch, four parameters are predicted to define the detection box: the x-coordinate (x) and y-coordinate (y) of the center point of the detection box, as well as its width (w) and height (h). The decoupling head mechanism operates as shown in Figure 3.

### 3.2. Loss Function

A critical factor in evaluating the model’s performance is its loss function. While YOLOv5 employs the CIoU loss function, CIoU fails to address the balance between positive and negative samples [21]. To overcome this limitation, Gevorgyan proposed the SIoU loss function, which redefines the penalty metric by incorporating the vector angles between adaptive weights and target regressions [22]. By leveraging the vector angle between regressions and adaptive weights, SIoU improves the accuracy of prediction boxes and enhances the overall detection performance of the system. This approach reduces the risk of overfitting or underfitting by effectively balancing positive and negative samples. The SIoU loss function primarily consists of four components: angular loss, shape loss, distance loss, and IoU loss, and is defined as follows:

(1) $$L_{SIoU}=1-IoU+\left(\Delta +\Omega \right)/2$$

where the shape loss and the distance loss are represented by Ω and Δ, respectively, SIoU incorporates the consideration of angular loss into the distance loss, as evidenced by the distance loss formula in Equation (2). The precise formulas for $\rho_{x}$, $\rho_{y}$, and γ are presented in Equation (3).

(2) $$\Delta =\sum_{t=x,y}\;(1-e^{-\gamma \rho_{t}})$$

(3) $$\rho_{x}={\left({(b}_{c_{x}}^{gt}-b_{c_{x}})/c_{w}\right)}^{2},\rho_{y}={\left({(b}_{c_{y}}^{gt}-b_{c_{y}})/c_{h}\right)}^{2},\gamma =2-\Lambda$$

where $c_{w}$ and $c_{h}$ are the width and height of the tiniest external rectangles that enclose both the ground truth and predicted boxes, respectively. Λ represents the angle loss, which is calculated as shown in Equation (4).

(4) $$\Lambda =1-2*sin^{2}\left(arcsin\left(c_{h}/\sigma \right)-\pi /4\right)$$

(5) $$\sigma =\sqrt{{\left(b_{c_{x}}^{gt}-b_{c_{x}}\right)}^{2}+{\left(b_{c_{y}}^{gt}-b_{c_{y}}\right)}^{2}}$$

(6) $$c_{h}=max\left(b_{c_{y}}^{gt},b_{c_{y}}\right)-min\left(b_{c_{y}}^{gt},b_{c_{y}}\right)$$

where *σ* symbolizes the gap, as determined by the given Equation (5), between the central point of the authentic ground box and the anticipated box. The height difference between the predicted box’s center and the ground truth box’s center is denoted by $c_{h}$, calculated as shown in Equation (6). The coordinates of the ground truth box’s center point (x, y) are $b_{c_{x}}^{gt}$ and $b_{c_{y}}^{gt}$. The coordinates of the center point of the prediction box’s center point (x, y) are represented by the variables $b_{c_{x}}$ and $b_{c_{y}}$. The shape loss is calculated using Equation (7). The precise formulas for $\omega_{w}$ and $\omega_{h}$ are presented in Equations (8) and (9), respectively.

(7) $$\Omega =\sum_{t=w,h}\;(1-e^{-\omega_{t}}{)}^{\theta }$$

(8) $$\omega_{w}=\left|w-w^{gt}\right|/\max\left(w,w^{gt}\right)$$

(9) $$\omega_{h}=\left|h-h^{gt}\right|/max(h,h^{gt})$$

where the prediction box’s height and width are indicated by h and w, respectively. The ground truth box’s dimensions are represented by $w^{gt}$ and $h^{gt}$. The degree of concern for form shape loss is represented by θ.

In the SIoU loss function, the mismatch penalty metric is evaluated between the predicted and ground truth bounding boxes, a task traditionally handled by conventional loss functions. While traditional approaches primarily focus on shape, IoU, and distance, SIoU introduces an additional consideration: the alignment between the ground truth box and the predicted box’s orientation. By incorporating direction as a key factor, SIoU reduces the overall degrees of freedom during optimization, enabling the predicted box to converge more rapidly toward the ground truth. This enhancement significantly improves both the inference speed and accuracy of the detection model in identifying anatomical structures of the fetal brain.

### 3.3. Target Filter

In contrast to natural image processing, a significant amount of prior medical information can be utilized in processing medical images [8]. This is especially true for object detection of anatomical structures in medical imaging, which requires analyzing each KAS in the image. In our fetal brain detection task, there are four standard planes and twelve anatomical structures, and these four planes are TLVAP, TTAP, TPFAP, and MSP. There are four anatomical structures in the TLVAP, which are CB, BM, and tow CPs; the TTAP contains four anatomical structures: CB, BM, AS, and T&P. There are four anatomical structures in the TPFAP: CB, CM, B, and FV. The MSP contains eight anatomical structures: CB, CM, B, FV, NB, M, TV, and HP. Except for the standard plane of the MSP, all other standard planes have four anatomical structures.

Algorithm 1 describes the filtering and sorting of anatomical structure detection results, removing duplicate and erroneous detection results and retaining the results with high confidence for each class of anatomical structures. Res_I = [Obj_1, Obj_2, Obj_3…, Obj_n], Obj_n = (x1, y1, x2, y2, conf, cls); the upper-left coordinate points of the object bounding boxes are indicated by x1, y1, and the lower-right coordinate points by x2, y2; conf refers to the object’s confidence in being detected, and cls stands for the object’s category. P_Objs is a dictionary that contains the target categories that are expected to be present in each standard plane. P_Objs = {TLVAP: [CB, BM, CP], TTAP: [CB, BM, AS, T&P], TPFAP: [CB, CM, B, FV], MSP: [CB, CM, B, FV, NB, M, TV, HP]}. Filtered_Res is derived from P_Objs to eliminate erroneous detection results, with an initial value of None. Best_Conf_Res retains the results from Filtered_Res that have a confidence level greater than 0.5 and selects the best result for each class, with an initial value of None. The prediction result for cp is referred to as Res_cp, and its initial value is None. Res_max represents the result with the highest confidence for each class, with an initial value of None. Res_now is the concatenation of Best_Conf_Res and Top_cp, with duplicates removed based on all columns, and its initial value is None. Top_cp is the best result selected from the top two records of Res_cp.

The model utilizes non-maximum suppression (NMS) to further refine object detection within the YOLO series. However, due to the complexity and variability of features in FBUS images, certain regions of the ultrasound image may exhibit characteristics resembling anatomical structures. This often leads to misidentification and the detection of redundant objects. To address this issue, we introduce a post-processing step based on medical prior knowledge, as outlined in Algorithm 1. This step involves filtering, reorganizing, and sorting the detection results to eliminate false positives and duplicate detections, ultimately retaining only the results with the highest confidence scores.
**Algorithm** **1.** An algorithm for filtering and sorting object detection results.**Require**: Res, P_Objs **Ensure**: Filtered_Res ← None, Best_Conf_Res ← None, Res_cp ← None, Top_cp ← None, Res_now ← None. 1: **Step 1:** Filter_results based on P_Objs 2: **for** result in Res **do** 3:      **if** result matches criteria defined by P_Objs **then** 4:          Filtered_Res ← Filtered_Res ∪ {result} 5:      **end if** 6: **end for** 7: **Step 2:** Retain results with conf > 0.5 and best per class 8: **for** each result in Filtered_Res **do** 9:      **if** result.conf > 0.5 **then** 10:        Best_Class_Res[result.class] ← Update with result ▷ Keep the best result per class 11:     **end if** 12: **end for** 13: **Step 3:** Retain top 2 results with the highest confidence for TLVAP with 2 CPs 14: **if ”CP**” in Res **then** 15:     Res_cp ← FilterResultsByNameCP(Res) 16:     Top_cp ← GetTopTwoByConfidence(Res_cp) 17: **end if** 18: **Step 4:** Sort, screen and rank the results 19: Res now ← ConcatenateAndRemoveDuplicates(Best_Class_Ress, Top_cp) 20: Res_now ← SortByAscending(Res_now, ’class’) 21: **return** Res_now

## 4. Experiments and Results

### 4.1. Experimental Setup

**Figure 4:**
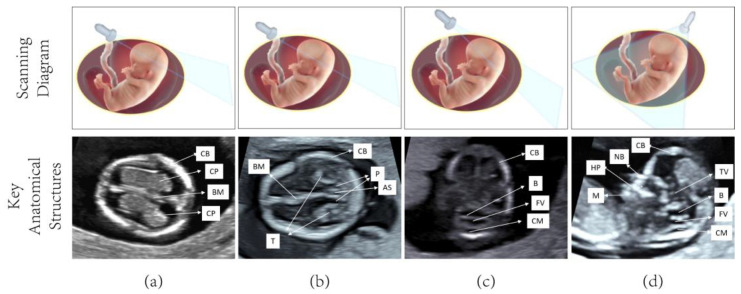
In these four subplots, the first row displays the clinical scanning diagram of the plane, while the second row showcases the ultrasound image of the plane along with its anatomical structures. (**a**) TLVAP; (**b**) TTAP; (**c**) TPFAP; (**d**) MSP.

Four standard ultrasound planes of the fetal brain were systematically collected during early pregnancy, with anatomical structures annotated for each plane as demonstrated in Figure 4 The dataset was developed in collaboration with clinical ultrasound specialists at two tertiary medical centers: the Quanzhou First Hospital and the Second Affiliated Hospital of Fujian Medical University. Following established clinical guidelines, ultrasound examinations were performed using high-frequency linear array probes (4–7 MHz) across eight advanced Doppler systems (IU22, IUELET, EPIQ5, EPIQ7 (Philips Healthcare, Best, North Brabant, The Netherlands); UGEO WS80A (Samsung, Medison Co., Ltd., Seoul, Republic of Korea); VolusonE6, VolusonE8, and VolusonE10 (General Electric Healthcare, Chicago, IL, USA). A total of 4086 FBUS images were acquired, capturing 1–3 distinct images per fetal plane to ensure anatomical variation representation. Among these images, the KASs were annotated in 2531 images by a professional sonographer, which were used for model training. The remaining 1573 unannotated images were set aside for testing the model’s performance, thereby enabling an evaluation of its generalization ability when encountering unseen data. The final dataset composition for fetal brain structure analysis is detailed in Table 1, showing the distribution between training and test sets.

The experiment was conducted using the following hardware configuration: an NVIDIA GeForce RTX 3050 GPU equipped with 16 GB of RAM (NVIDIA Corporation, Santa Clara, CA, USA), a 12th Generation Intel^®^ Core™ i5-12400F CPU (Intel Corporation, Santa Clara, CA, USA). The operating system was Windows 10. The programming environment utilized was Python 3.8.18, and the deep learning framework employed for the experiment was PyTorch 2.1. The optimizer, learning rate, and other test parameters were configured based on YOLOv5. Specifically, the learning rate was set to 0.001, and the weight decay was also set to 0.001. The Intersection over Union threshold was configured at 0.41, while the anchor threshold was set to 5.0. The model was trained for 300 epochs, with an input image size of 640 × 640 pixels. The Stochastic Gradient Descent optimizer was used for training. These parameters were carefully selected to optimize the model’s performance.

### 4.2. Evaluation Metrics

The detection performance was evaluated using Precision, Recall, mAP@0.5, and mAP@0.5:0.9, as specified in the Formulas (10), (11), (12), and (13), respectively. In Equations (10) and (11): TP: The model correctly detects fetal brain anatomical structures. FP: The model falsely detects non-existent structures. TN: The model correctly excludes non-target regions. FN: The model misses actual anatomical structures. These symbols are used to compute the metrics mentioned above. The area under the P–R curve is called the Average Precision, or AP for short. A higher AP value indicates better model performance. The mean Average Precision, or mAP for short, is the average value of AP for each category. One of the most crucial evaluation metrics for object detection tasks is the mAP, whose values range from 0 to 1. The higher the mAP, the better the model’s performance. When the IoU is set to 0.5, mAP@0.5 is used to calculate mAP; when the IoU threshold is between 0.5 and 0.95 (step size of 0.05), mAP@0.5:0.95 is used to calculate mAP.

(10) Precision=TP/(TP+FP)

(11) Recall=TP/(TP+FN)

(12) $$AP=\int_{0}^{1}P(r)dr$$

(13) $$mAP=\sum_{i=1}^{n}AP_{i}/n$$

For evaluating our model’s complexity, we employed the number of parameters as a typical metric used in deep learning models. Nevertheless, given the typically vast number of parameters in such models, we typically resort to megabytes (MB) as the unit of quantification. Turning our attention to the inference speed, we aimed to minimize the potential for accidental errors by selecting a subset of 600 FBUS images from the test set and permitting the model to perform inference on the GPU. Following this, we determined the inference speed for each image by averaging the time taken by the model to process each of the 600 images. Additionally, to ensure that our results more closely reflect the model’s performance in real-world scenarios, we configured the batch size to 1 when utilizing the target detection models for inference on FBUS images.

### 4.3. Experiment Results

#### 4.3.1. Detection Results

**Figure 5:**
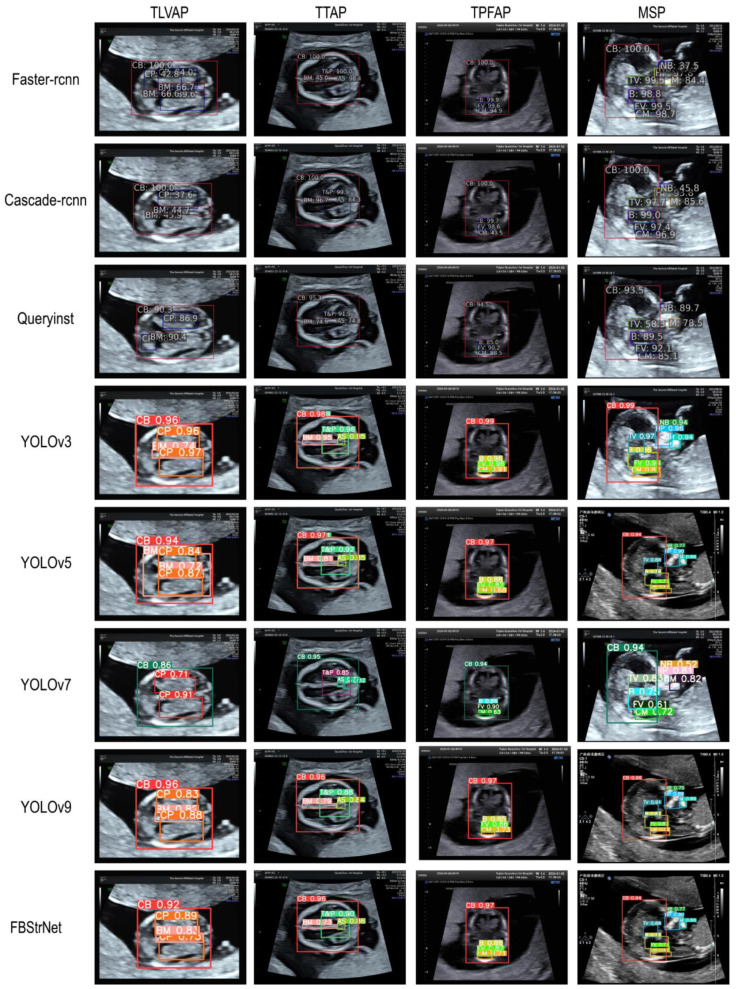
The visualization of several models that perform well in the detection task of FBUS images, as well as the detection results of our model FBStrNet.

**Figure 6:**
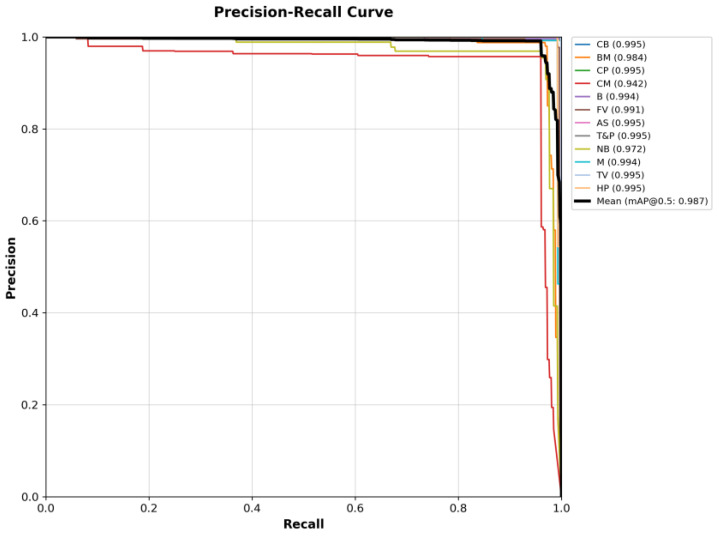
FBStrNet’s P–R curve.

During the object detection task, we compare our model with the currently better-performing object detection models, such as YOLOv3 [23], YOLOv5 [24], YOLOv7 [25], YOLOv9 [26], Cascade R-CNN [27], QueryInst [28], and Faster R-CNN [29]. The performance metrics presented in Table 2 demonstrate that our FBStrNet model achieves superior performance across all metrics for the FBUS anatomical structure detection task. Among the compared models, YOLOv9 stands out as the top performer for this task. Our model exceeds YOLOv9 by a marginal 0.3% improvement in precision, while simultaneously exhibiting significantly fewer parameters and delivering faster inference speeds compared to YOLOv9. In Figure 5, it presents the results obtained by several well-performing models for FBUS image detection. The portrayed results clearly illustrate that FBStrNet excels at accurately and effectively identifying each KAS within an FBUS image. To further assess the recognition capabilities of FBStrNet across various anatomical structures, we have plotted the P–R curves on the test set, as illustrated in Figure 6. Furthermore, it is worth noting that our proposed approach enables rapid identification and detection of FBUS images within a mere 11.5 ms, making it highly suitable for real-time clinical scanning requirements during fetal brain examinations, where sonographers can conveniently visualize anatomical structures in ultrasound images.

#### 4.3.2. Model Visualization Results

**Figure 7:**
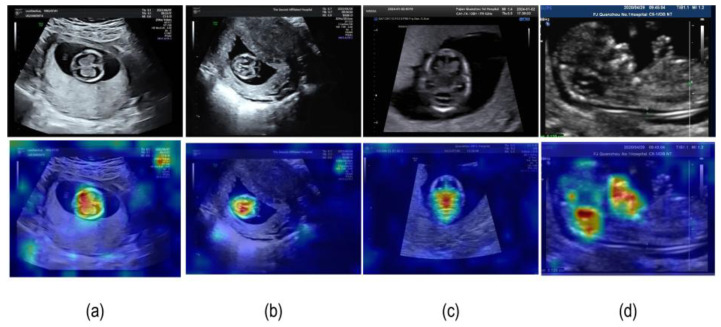
The Grad-CAM visualization results of FBStrNet. (**a**) TLVAP; (**b**) TTAP; (**c**) TPFAP; (**d**) MSP.

Grad-CAM (Gradient-weighted Class Activation Mapping) [30] has emerged as a robust visualization technique for interpreting deep learning models in classification tasks. This method generates class activation maps by computing gradients from the final convolutional layer, enabling the identification of critical image regions that significantly influence the model’s classification outcomes. By highlighting class-specific discriminative features, Grad-CAM provides valuable insights into the model’s decision-making process. In the current study, we implemented Grad-CAM to precisely localize and visualize regions of interest within fetal ultrasound images, as demonstrated in Figure 7.

### 4.4. Ablation Experiments

To evaluate the effectiveness of our proposed model in identifying fetal brains, we performed ablation studies targeting Ghost, the SIoU loss function, and Decoupled_Detect. The hyperparameter settings were kept constant during the ablation experiments performed. The results of these experiments are summarized in Table 3, which highlights the benefits of various model-structure optimization techniques. Notably, FBStrNet achieved a 0.4% boost in mAP@0.5 while maintaining the same number of parameters compared to the baseline YOLOv5 model.

First, we test the performance of each module on a baseline basis. The Ghost module reduces the number of parameters to 5.1 M, slightly increases the inference time to 10.0 ms, and marginally improves mAP@0.5 and mAP@0.5:0.95 to 98.4% and 64.6%, respectively. The SIoU loss function significantly enhances mAP @0.5 and mAP@0.5:0.95 to 98.9% and 65.6% while maintaining the same number of parameters and inference time. Although the Decoupled_Detect module increases the number of parameters to 8.8 M and extends the inference time to 11.6 ms, it also significantly improves mAP@0.5 and mAP@0.5:0.95 to 98.8% and 66.4%, respectively. Next, building upon the baseline model, we introduced the Ghost and SIoU modules, which reduced the parameter count to 5.1 M while maintaining an inference time of 10.0 ms. While this configuration resulted in a slight improvement in mAP@0.5, it led to a decline in mAP@0.5:0.95. To further enhance performance, we integrated the Ghost, SIoU, and Decoupled_Detect modules into the baseline model. This combination increased the parameter count to 6.8 M and extended the inference time to 11.5 ms, but it achieved notable improvements in both mAP@0.5 and mAP@0.5:0.95 metrics.

The SIoU and Decoupled modules demonstrate remarkable effects in enhancing accuracy, particularly for the mAP@0.5:0.95 metric. The Ghost module effectively reduces the number of parameters with minimal impact on inference time. The combination of Ghost, SIoU, and Decoupled achieves a good balance between accuracy and parameter count.

To evaluate the impact of different loss functions on the performance of FBStrNet, we conducted experiments using both CIoU and SIoU as the loss functions. The results are presented in Table 3. When compared with the CIoU loss function, the SIoU loss function brings about a significant improvement in mAP@0.5, with an increase of 0.5%. However, there is a minor decrease of 0.1% in mAP@0.5:0.95. In terms of model complexity, the number of parameters reduces by 0.1 M when using the SIoU loss function. On the other hand, the inference time increases by 0.3 ms.

## 5. Discussion

This study proposes the FBStrNet model, which can effectively detect anatomical structures in fetal brain ultrasound images during early pregnancy. Experimental results demonstrate that it significantly outperforms existing methods in detection accuracy. FBStrNet builds upon YOLOv5 as the baseline model, incorporating a series of improvements tailored for detecting key anatomies in fetal brain ultrasound images. The selection of YOLOv5 as the baseline model is justified by several advantages: (1) YOLOv5 has a smaller number of parameters compared to other models (as shown in Table 2), making it easier to deploy and run on resource-constrained devices, such as hospital equipment; (2) YOLOv5 offers faster inference speeds, which is critical for real-time clinical scanning; and (3) YOLOv5 provides high flexibility, allowing us to adapt it to our specific data characteristics and requirements.

The filtering logic of Algorithm 1 strictly relies on the predefined target categories in P_Objs, which effectively reduces false positives. Still, it simultaneously limits its ability to detect new or undefined categories, leading to false negatives, especially when encountering unlisted anatomical structures such as rare pathological variants. Additionally, the accuracy of P_Objs directly impacts the algorithm’s performance; if the definitions are incomplete, the filtering effectiveness is significantly compromised. The use of a fixed confidence threshold (e.g., 0.5) to filter results lacks flexibility, making it difficult to adapt to differences in categories and image quality. This approach can easily result in the erroneous filtering of true targets in low-quality images, while in high-quality images, artifacts or noise may cause false positives. Therefore, it is necessary to introduce a dynamic category expansion mechanism, integrating unsupervised learning or anomaly detection to identify new categories, and adopt a dynamic threshold mechanism that automatically adjusts the confidence threshold based on image quality and category characteristics to enhance the algorithm’s adaptability.

Previous studies have primarily focused on the research of fetal brain ultrasound images obtained during the second or third trimester of pregnancy [11,12,13,14,15,16,31,32,33,34,35,36]. In these ultrasound images, anatomical structures form a distinct contrast with the surrounding tissues, facilitating clear observation and detection. In contrast, this study shifts its focus to ultrasound images of the fetal brain in the first trimester, particularly TLVAP, TTAP, TPFAP, and MSP. As shown in Figure 6, compared with other structures, FBStrNet has a lower mAP@0.5 in identifying CM and NB. The reason is that these two anatomical structures have lower contrast with their surrounding tissues, which makes identification difficult. Research on the fetal brain in the first trimester has mainly concentrated on measuring the crown-rump length and nuchal translucency within the MSP plane, which are macroscopic indicators [1,14,17,18]. However, FBStrNet exhibits unique potential, as it can comprehensively detect anatomical structures across multiple planes. To fully leverage this capability, future work should prioritize the adoption of image processing techniques that can enhance the clarity of ultrasound images, such as noise reduction and contrast adjustment, thereby improving the model’s ability to distinguish minute structures.

## 6. Conclusions

This study introduces FBStrNet, a model for real-time, accurate detection of key structures in fetal brain ultrasound images, aiding clinicians in rapid screening for cranial abnormalities. Results show FBStrNet effectively locates critical structures, improving detection accuracy and efficiency. It serves as a valuable tool for both clinical diagnosis and medical education. However, its performance is limited by dataset size and diversity. Future work will involve collaborating with hospitals to expand datasets and explore image quality assessment methods to enhance robustness. In summary, FBStrNet offers a practical solution for fetal brain analysis, with significant clinical potential and a foundation for further research.

## Figures and Tables

**Table 1:** The number of anatomical structures contained in the training set and the test set.

	Train	Test
CB	2518	1866
BM	1251	1010
CP	1266	938
CM	1267	856
B	1267	856
FV	1267	856
AS	618	539
T&P	618	539
NB	649	317
M	649	317
TV	649	347
HP	649	347

**Table 2:** The results of each model’s identification of the FBUS’s anatomical structure.

Model	Precision	Recall	mAP@0.5	mAP@0.5:0.95	Parameters (M)	Times (ms)
YOLOv3	98.6%	98.6%	98.3%	66.4%	61.5	30.9
YOLOv5-n	98.7%	98.5%	98.3%	65.8%	1.8	8.4
YOLOv5-s	98.2%	97.4%	98.3%	64.2%	7.0	11.2
YOLOv7	94.8%	95.1%	96.4%	56.5%	36.5	65.4
YOLOv7-x	95.5%	96.6%	96.8%	58.3%	70.9	53.1
YOLOv9-c	98.3%	98.5%	98.8%	69.0%	60.8	58.5
YOLOv9-e	98.2%	98.3%	98.5%	69.3%	69.5	62.4
Faster R-CNN	-	-	91.4%	57.8%	41.4	56.1
Cascade R-CNN	-	-	92.0%	59.8%	69.4	74.5
QueryInst	-	-	96.1%	65.9%	172.5	121.0
FBStrNet (Ours)	98.6% (+0.3%)	98.5%	98.7%	64.3%	6.9	11.5

**Table 3:** The results of ablation tests conducted using Ghost, SIoU, and Decoupled_Detect in the FBStrNet.

Ghost	SIoU	Decoupled_Detect	mAP@0.5	mAP@0.5:0.95	Parameters (M)	Time (ms)
Baseline(YOLOv5-s, CIoU)	×	×	98.3%	64.2%	7.0	9.8
√	×	×	98.4%	64.6%	5.1	10.0
×	√	×	98.9%	65.6%	7.0	9.8
×	×	√	98.8%	66.4%	8.8	11.6
√	√	×	98.5%	63.1%	5.1	10.0
√	×	√	98.2%	64.4%	6.9	11.3
√	√	√	98.7%	64.3%	6.8	11.5

## Data Availability

The data are not accessible to the public owing to patient privacy concerns. If you have any research needs, you can contact the corresponding author for assistance.
